# Removal of Cyanobacteria and Cyanotoxins in Waters

**DOI:** 10.3390/toxins13090636

**Published:** 2021-09-09

**Authors:** Albert Serrà, Laetitia Philippe, Elvira Gómez

**Affiliations:** 1 Thin Films and Nanostructures Electrodeposition Group (GE-CPN), Department of Materials Science and Physical Chemistry, University of Barcelona, Martí i Franquès 1, E-08028 Barcelona, Catalonia, Spain; 2 Institute of Nanoscience and Nanotechnology (IN2UB), Universitat de Barcelona, E-08028 Barcelona, Catalonia, Spain; 3 Manufacture des Montres ROLEX SA, Research & Development, CH-2501 Bienne, Switzerland; laetitia.philippe@gmail.com

Harmful cyanobacterial algal blooms and cyanotoxins currently pose a major threat to global society, one that exceeds local and national interests due to their extremely destructive effects on the environment and human health. In the near future, the formation of harmful cyanobacterial algal blooms and, in turn, cyanotoxins is expected to become widespread, driven by eutrophication and anthropogenic causes such as water pollution and promoted by escalating global temperatures. Such trends, studied since the late 1990s, have attracted increased interest due to (i) the high environmental impact of cyanobacterial blooms and cyanotoxins worldwide, (ii) the ineffective removal of those pollutants by conventional water treatment processes, (iii) the transformational capacity to completely destroy those organic toxins via alternative treatments such as advanced oxidation processes, and (iv) engineering challenges when transitioning toward the treatment of large volumes of water. The global context of this threat thus urges the innovation of simple, sustainable, low-cost strategies and technologies for water decontamination that can be readily implemented worldwide [1].

In this Special Issue, titled “Removal of Cyanobacteria and Cyanotoxins in Waters,” we have attempted to provide readers with a comprehensive overview of different strategies and alternative treatments currently being studied for the effective removal of cyanobacteria and cyanotoxins from water. The following presents a brief synopsis of the 13 research papers that constitute this Special Issue.

Coagulation is a key process employed by conventional water treatment technologies. Worldwide, the most widely applied water treatment technology at water treatment plants involves a combination of coagulation, flocculation, sedimentation, filtration, and disinfection to treat water. Herein, five papers focus either directly or indirectly on coagulation and its alternatives or on the potential impacts of conventional water treatment technologies on drinking water containing cyanobacteria and/or cyanotoxins [2]. Arruda et al. [2] have demonstrated that the so-called “floc-and-skin” technique, based on the combination of a ballast (i.e., natural soil or modified clay) and a coagulant, can effectively remove cyanobacterial biomass comprised of *Dolichospermum circinalis* and *Microcystis aeruginosa*, depending on the ballast’s capacity to adsorb microcystin. At the laboratory scale, the authors studied the effect of the floc-and-skin technique on the release of microcystins from cyanobacterial biomass in real water from the Funil Reservoir, a eutrophic system in southern Brazil. Their results demonstrate that the technique is more effective than using a sole coagulant (i.e., polyaluminum chloride) to remove cyanobacteria and extracellular microcystins from water [2]. Next, Mucci et al. [3] studied the effect of using chitosan as a coagulant to remove cyanobacteria from the water column. Although chitosan’s coagulation efficiency has been extensively studied, little is known about its effect on the viability of cyanobacteria cells and the release of cyanotoxins. The authors’ study confirmed that chitosan was able to damage *Microcystis aeruginosa* cells by inducing lysis and, consequently, the release of cyanotoxins, which thus inhibit chitosan’s use in lake restoration without directly testing its effects on the natural target biota because, as the authors show, the side effects depend on the strain [3]. Lürling et al. [4] also examined the efficiency of deploying the floc-and-skin technique to control the growth of cyanobacterial blooms using a combination of a coagulant (i.e., polyaluminum chloride) and a ballast (i.e., lanthanum modified bentonite) from Lake De Kuil and Lake Rauwbraken in the Netherlands. Above all, the authors demonstrate the necessity of testing the technique’s process in the water to be treated, for the technique, when used with similar species of *Planktothrix rubescens,* yielded different results depending on the lake. After one day, the filaments of a *Planktothrix rubescens* biomass from Lake De Kuil resurfaced but remained precipitated in Lake Rauwbraken, possibly due to water matrix effects. By contrast, Pinkanjananavee et al. [5] studied the effectiveness of using conventional treatment processes consisting of coagulation, flocculation, sedimentation, filtration, sludge dewatering, and disinfection to remove *Microcystis aeruginosa* and microcystin-LR prior to disinfection. Noting that several conventional drinking water treatment plants recycle dewatered supernatant from sludge-dewatering operations, the authors aimed to determine the impact of recycling dewatered sludge supernatant on the quality of treated water in laboratory-scale experiments. Ultimately, their study demonstrated that recycling dewatered sludge supernatant can indeed affect the quality of treated water. The fifth paper, by Jalili et al. [6], describes the effects of various cyanobacteria in a treatment plant for drinking water before, during, and after the occurrence of cyanobacterial blooms by monitoring raw water, sludge in the holding tank, and the sludge supernatant. In view of their results, the authors conclude that predicting the cyanobacterial community in the sludge supernatant continues to be a challenge.

Three other papers address different advanced oxidation processes used to remove cyanotoxins [7,8,9]. Advanced oxidation processes involve a set of oxidative water treatments, including treatment with UV–O_3_, UV–H_2_O_2_, the Fenton process, the photo-Fenton process, and nonthermal plasmas as well as sonolysis, photocatalysis, and radiolysis, all based on the production of powerful, highly reactive oxidants. Among those oxidants, hydroxyl radicals are the most commonly used and are the strongest known oxidants after fluorine, and, similar to other highly reactive oxidants, can nonselectively destroy the majority of organic matter and can mineralize organic pollutants in water. Against that background, Ferreira et al. [7] investigated the applicability of the Fenton process to mineralize cylindrosmpermopsin by analyzing how the dosage of Fenton reagents, namely H_2_O_2_ and Fe(II), and the H_2_O_2_-to-Fe(II) molar ratio affected the removal of cylindrosmpermopsin in ultrapure water (i.e., unrealistic conditions that do not consider water matrix effects or real pH) [7]. Although the Fenton process is a promising advanced oxidation technique that can be easily implemented at full scale worldwide due to its simplicity and highly cost-effective technology, the water matrix effect and large-scale volumes need to be investigated to evaluate the process for application in treating cyanotoxins because the presence of scavengers and competing species identified in real water matrices may affect degradation kinetics. Next, Benamara et al. [8] report that Al-doped ZnO nanoparticles are effective photocatalysts for degrading and mineralizing two typical cyanotoxins, microcystin-LR and anatoxin-A, under visible-light irradiation with light emitting diodes (LEDs). Central to that process is using easily fabricated Al-doped ZnO nanoparticles to act as photocatalysts for water decontamination. Their paper, describing the visible-light-driven removal of cyanotoxins in the presence of ZnO-based photocatalysts, shows that high removal efficiencies can be achieved under simple conditions. The authors also found that the system’s outstanding performance derives from the excellent photocatalytic performance and the high chemical and photochemical stability of Al-doped ZnO nanoparticles under visible-light irradiation with LEDs [8]. In another study, Sorlini et al. [9] investigated the viability of using UV–H_2_O_2_ to treat microcystin-LR, particularly by analyzing specific energy consumption in the waters of Lake Iseo in the Province of Brescia, Lombardy, Italy. Those authors aimed to preliminarily study the effectiveness of UV–H_2_O_2_ on real water with respect to water matrix effects in order to clarify how the type of oxidant influences kinetics by analyzing the effects of the initial microcystin-LR concentration and the H_2_O_2_ dosage. The authors also examined the total specific energy consumption of UV–H_2_O_2_ compared to UV treatment to determine the optimal operational conditions [9]. Although the authors suggest the potential of using those three advanced oxidation processes to remove various cyanotoxins, unrealistic laboratory settings and conditions that allow the treatment of small volumes of water with highly energy-intensive lamps underscore some of the challenges of applying those technologies in the near future.

Because different chemical oxidants can be used to treat water, the effects of some of those oxidants are discussed in three additional papers. In one of those papers, because H_2_O_2_ is a highly investigated Fenton reagent used in different water treatment strategies, Lusty et al. [10] investigated the effects of H_2_O_2_ on cyanobacteria and microbial communities in order to analyze its use to mitigate potentially toxic freshwater cyanobacterial blooms. The study revealed that H_2_O_2_ can effectively reduce but not fully eliminate cyanobacteria from eutrophic bodies of water; thus, the authors conclude that H_2_O_2_ is not the best candidate for use in high biomass ecosystems containing harmful cyanobacterial algal blooms. By comparison, Moradinejad et al. [11] investigated variation and shifts in the structure of cyanobacterial communities during chemical oxidation (i.e., with Cl_2_, KMnO_4_, O_3_, and H_2_O_2_) using the metagenomic shotgun approach, which allows the consideration of the diversity of cyanobacterial communities in pre-oxidant selection in the on-site management of cyanobacterial blooms. According to their results, only pre-oxidation with H_2_O_2_ exhibited a clear decrease in the abundance of cyanobacterial biomasses, and the authors add that the selection of the pre-oxidant translates into considerable reductions in cost. Beyond that, the paper by Greenstein et al. [12] provides guidance on how five oxidants (i.e., Cl_2_, NH_2_Cl, ClO_2_, KMnO_4_, and O_3_) affect the delayed release of intracellular microcystins after the partial oxidation of cyanobacteria as a critical parameter to consider, especially for drinking water treatments, because the concertation of cyanotoxins in water can increase after several hours of treatment.

From another angle, El Amrani Zerrifi et al. [13] investigated the potential of employing natural compounds extracted from seaweeds to control harmful algae in aquatic ecosystems. The authors report the results of a series of anti-cyanobacterial assays with different extracts from seaweeds from Morocco, which demonstrate the anti-cyanobacterial properties of *Cystoseira tamariscifolia* and its potential use in developing environmentally friendly procedures to control the growth of toxic cyanobacteria.

Last, the paper by Esterhuizen [14] presents a systematic approach to planning, constructing, monitoring, and optimizing a phytoremediation system for the treatment of wastewater from aquaculture. In their study, the authors constructed a large-scale Green Liver System requiring minimal maintenance and low construction costs based on the use of macrophytes. Because aquacultural wastewaters are eutrophic, cyanobacterial blooms flourish in them and can often result in the presence of cyanobacterial toxins. However, the authors’ study demonstrated that various microcystins were effectively removed in their bioremediation process.
